# Multitasking: does task-switching add to the effect of dual-tasking on everyday-like driving behavior?

**DOI:** 10.1186/s41235-025-00611-y

**Published:** 2025-02-08

**Authors:** Piesie A. G. Asuako, Robert Stojan, Otmar Bock, Melanie Mack, Claudia Voelcker-Rehage

**Affiliations:** 1 Department of Neuromotor Behavior and Exercise, Institute of Sport and Exercise Sciences, University of Muenster, Muenster, Germany; 2 Institute of Exercise Training and Sport Informatics, German Sport University, Cologne, Germany; 3 University of Geneva, Geneva, Switzerland

**Keywords:** Dual-tasking, Task-switching, Driving simulator, Ecological validity, Young adults, Motor task, Cognitive task

## Abstract

It is well established that performing multiple tasks simultaneously (dual-tasking) or sequentially (task-switching) degrades performance on one or both tasks. However, it is unknown whether task-switching adds to the effects of dual-tasking in a single setup. We investigated this in a simulated everyday-like car driving scenario. We expected an additive effect of task-switching on dual-tasking, leading to a stronger deterioration of driving performance due to the increased cognitive load required to handle multiple task-sets. Forty-five young adults aged 18 to 30 years (age: 23.62 ± 2.51, 28 females) were instructed to follow a lead car driving at a constant speed of 70 km/h through a rural landscape while concurrently performing additional tasks. The additional tasks were typing and arguing, in response to stimuli presented visually or auditorily. The tasks were presented either in separate blocks or in intermixed order (conditions: repetitive vs. switching). Driving performance was assessed by use of the average velocity and the standard deviation of lateral position, and performance in the additional tasks was assessed by reaction time. Linear-mixed effect models revealed better performance in the repetitive, compared to the switch condition only for the standard deviation of the lateral lane position while performing the additional typing task. This provides limited evidence for the view that task-switching adds to the challenges of dual-tasking. We therefore posit that already dual-tasking alone involves processing demands that are not substantially increased by adding switching demands.

**Supplementary Information:**

The online version contains supplementary material available at 10.1186/s41235-025-00611-y.

## Public significance statement

In many everyday situations, we perform two tasks concurrently (dual-tasking) or alternately (task-switching). One example is driving while talking to a passenger or using the board computer (in-vehicle infotainment system). Our study shows that driving while concurrently performing an additional task is similar regardless whether the additional task is always the same or whether it differs (task-switching). Thus, although the additional task itself (typing versus thinking) might influence driving results, the order of the tasks does not.

## Multitasking: does task-switching add to the effect of dual-tasking on everyday-like driving behavior?

Multitasking is a common everyday behavior that can occur in various ways, such as when performing two or more tasks at the same time or performing one task shortly after another. For example, preparing dinner in the kitchen while attending to the playful baby in the living room, driving while talking to a passenger, or walking while interacting with a mobile phone are common activities in everyday life. In scientific literature on multitasking, performing two tasks at the same time is described as dual-tasking (Hazeltine et al., 2006), while completing two tasks in short temporal succession is defined as task-switching (Koch et al., 2018). In everyday life, people engage in various dual-tasks, while also often switching between them, i.e., task-switching while dual-tasking. For instance, in a situation where a driver is talking to a passenger, then adjusts the in-vehicle stereo, and then returns to the conversation with the passenger while driving continuously. In this scenario, the driver switches back and forth between two different dual-tasks, i.e., driving while talking to a passenger and driving while adjusting in-vehicle stereo. In the scientific literature, it has been established that both dual-tasking and task-switching independently lead to performance deterioration in either one or both tasks (Hazeltine et al., 2006; Kiesel et al., 2010; Koch et al., 2018). This raises an important question: Does the effect of task-switching further add to the challenges of dual-tasking when both occur within the same scenario? Or more specific, in the driving example above, does the driver’s performance further deteriorate when switching between the two dual-tasking scenarios? Answering this question is essential for multitasking research, in particular, when aiming to effectively transfer the basic mechanisms of dual-tasking and task-switching observed from controlled laboratory studies toward experimental paradigms that mimic real-life conditions. This study investigates the effects of task-switching on dual-task performance (i.e., driving and additional task performance) in an everyday-like virtual driving scenario.

Several studies already have investigated dual-tasking under everyday-like conditions utilizing advanced technology such as virtual reality, which allows to expand laboratory research by using more ecologically valid paradigms, without giving up the advantages of a laboratory setting such as controllability, standardization, and safety (Calhoun & Pearlson, 2012). For instance, Drews et al. (2009) and Horrey and Wickens (2004) investigated dual-tasking (text messaging, phone number task) during simulated car driving and showed that drivers’ responses to unexpected road hazards were impaired under dual-tasking conditions compared to driving-only conditions (single-tasking). Other studies, such as Byington and Schwebel (2013) and Neider et al. (2010), examined dual-tasking in the context of simulated street crossing while engaging in manual tasks involving mobile phone or other comparable numerical devices, respectively. Both studies demonstrated that pedestrians tended to wait longer to cross and missed more safe opportunities to cross the street when engaged in dual-tasking during street crossing. A common feature of the experimental design in both studies was that the additional task was repeated within the dual-tasking condition. However, in real life, we often switch between different additional tasks or between different dual-task conditions. Only a few other studies (Bock et al., 2021; Janouch et al., 2018; Stojan & Voelcker-Rehage, 2021; Stojan et al., 2024; Wechsler et al., 2018) have introduced an intermixed presentation of additional tasks while participants simultaneously performed the primary task, like car driving or walking. However, these studies did not investigate whether switching between different additonal tasks compounded their observed effects of dual-tasking on driving or street crossing performance. To the best of our knowledge, no study has investigated yet the potential effect of task-switching on dual-tasking within a single setup, neither in a laboratory nor a more everyday-like experimental setting. Thus, in the current study, we investigated the effect of task-switching on dual-task performance in an everyday life scenario, using car driving as an example.

In recent studies by Bock et al. (2021) and Stojan et al. (2024), participants performed a braking maneuver task under two conditions. In the multitasking condition, participants completed the braking task alongside an intermixed sequence of additional tasks (i.e., the additional tasks were presented in a mixed order rather than presented in a predictable pattern), while in the control condition, they performed the braking task without presence of additional tasks. Results indicated that the effects of additional tasks on braking responses persisted for up to 11.5 s in the multitasking condition, providing initial evidence for an additional effect of task-switching on dual-tasking costs. Several mechanistic explanations target this effect: a short-lived psychological refractory period (PRP) effect, which suggests that due to the central processing stage’s inability to deal with parallel tasks, the second task must wait until the first task is processed, creating a “central bottleneck” that extends the reaction time (RT) for the second task (Pashler, 1994; Pashler et al., 2008; Welford, 1952). However, the PRP effect is rather short-lived. Studies have shown that when the stimulus onset asynchrony (SOA) between tasks increases to about 400 ms, the RT in the second task decreases and remains steady as SOA increases beyond 400 ms and approaches 1 s (Hibberd et al., 2010; Karlin & Kestenbaum, 1968; Levy et al., 2006). A more persistent influence might be associated with a "task-set" effect from prior additional tasks. Task-set effect refers to the cost associated with maintaining, switching or managing specific set of rules, goals and responses relevant to task, especially when multiple tasks or task rules are involved (Koch et al., 2018). As the aforementioned studies by Bock et al. (2021) and Stojan et al. (2024) also suggest, switching between different additional tasks while driving may have required participants to maintain and reconfigure multiple task-sets. In task-switching research, the persistent task-set effect is explained by two main theories: the task-set inertia theory (Allport & Wylie, 1999, 2000) and the task-set reconfiguration theory (Rogers & Monsell, 1995). Task-set inertia reflects the lasting activation of a previous task-set in memory that must be inhibited in order to select, retrieve, and implement a new task-set (Allport & Wylie, 1999, 2000). This theory suggests that switching from an old task to a new one requires the decay of the now-irrelevant task-set in memory, which slows down the news task’s performance. In a slightly different theoretical approach, the task-set reconfiguration theory assumes that a task-set that is no longer relevant needs to be replaced with a new task-set that is relevant to the current task (Rogers & Monsell, 1995). According to this theory, the time spent removing the task-set for the previous task and replacing it with the task-set of the new task describes the observed switch costs. However, once the task-set of the previous task is removed, there is no carry-over effect on the new task (Grange & Houghton, 2014).

In comparison with the previous studies of Bock et al. (2021) and Stojan et al. (2024), the present study aimed to investigate whether switching between different dual-tasks leads to performance decrements compared to performing the same dual-tasks repetitively in an everyday-like virtual driving scenario. Hence, a driving simulator setup was used to address this research question in a controlled everyday-like virtual environment. In this study, a task-switching condition, in which additional tasks, i.e., typing task (TYPE) and argumentation task (ARG) during driving were presented in an intermixed order (to replicate the ever-changing sequences of real life) was compared with a condition in which additional tasks during driving were presented block-wise (simulating conventional dual-task paradigms). Based on the persistent task-set theory, we hypothesized that the persistent task-set effect from the previous dual-task would compound the performance of the new dual-task. Specifically, we expected worse driving performance in the condition, where tasks were presented in an intermixed order (task-switching while dual-tasking), compared to the condition where tasks were presented in a repetitive order (dual-tasking only). Additionally, we explored whether switching between different dual-tasks varied based on the specific task demands by further examining differences between task types (TYPE or ARG) and presentation modalities auditory (aud) or visual (vis). To date, the question whether switching between different dual-tasks is further influenced by the specific demands of the dual-task-type(s), such as the duration of the information processing stage of each task, remains unclear. Therefore, in the task-switching condition, and based on the persistent task-set theory, we hypothesized that due to the longer processing duration of the a ARG, particularly when presented in the aud modality, switching from an ARG to a TYPE would result in a stronger task-set effect on driving performance and RT of additional task than switching from a TYPE to an ARG, especially in the vis modality that typically is more interfering with the visuomotor demands of car driving.

The result of this study may provide further insights into the interaction of dual-task and task-switching in our everyday-like activities such as car driving. Our findings may reveal that extra mental effort and cognitive control is required to maintain performance due to the concurrence of both dual-tasking and task-switching compared to dual-task alone.

## Methods

### Participants

This study forms part of a larger research project on multitasking (training-induced plasticity of multitasking in everyday-like motor behavior, German Research Foundation (DFG) Priority Program, SPP 1772). An a priori power analysis (Faul et al., 2007) was performed with G*Power 3.1.9 software to determine the sample size to answer our main hypothesis, i.e., multitasking deficits will be greater when tasks are performed intermixed. For an ANOVA (α = 0.05, ß = 0.80, *f* = 0.20, number of groups = 1, number of measurements = 2, *r* (condition) = 0.7), the analysis revealed that a sample size of 32 participants was sufficiently high to detect the expected effect. To account for an attrition of 20%, we aimed to recruit at least 45 young adults for our experiment. They were recruited via posters at public places, the university’s weekly newsletter and students’ mailing list. Participants were included in the study if they: (a) were aged between 18 and 30 years, (b) possessed a valid driving license, (c) were regular drivers, that is, drove at least once every week during the last 6 months, (d) had no acute or chronic physical, cognitive, or neurophysiological condition (self-reported), such as previous strokes, brain injuries, or heart attacks. Persons who usually wore glasses while driving did so during the study. In total, 45 young adults between 18 and 30 years (age: 23.62 ± 2.51, females = 28) participated in this study.

Two participants dropped out of the study without providing reasons. Due to technical issues with the recording of verbal data, eleven other participants had to be excluded from the RT analysis (i.e., additional tasks), but retained for driving behavior analysis. As a result, 43 participants were included in the driving behavior analysis, while 32 participants were included in the additional task analysis (cf. Table 1 for demographics of the final sample). Participants were compensated with either 20€ or course credit points. Before participating in the study, all participants signed informed written consent. The study procedure was approved by the ethics committee of the University of Chemnitz, Germany (registration number V-280–17-CVR-Multitasking-29062018), and was conducted in compliance with the Declaration of Helsinki (World Medical Association, 2014).

**Table 1:** Participants’ demographic information

	Total participants* (N*= *45) M(SD)*	Included in driving behavior analysis* (n* = *43) M (SD)*	Included in additional task analysis* (n* = *32) M (SD)*	Test statistic (*X*^*2*^* or H*)	*p*
Age	23.62 (2.51)	23.52 (2.48)	23.53 (2.32)	*H* = 0.04	0.98
Sex (M|F)	17|28	17|26	13|19	*X*^*2*^ = 0.02	0.99
Education	16.28 (2.30)	16.25 (2.27)	16.53 (2.28)	*H* = 6.11	0.05
Distance traveled per week (km)	69.21 (85.55)	67.98 (84.09)	65.31 (69.72)	*H* = 1.78	0.41

Mean (*M*) and standard deviation (*SD*) of age are presented. Sex is presented in absolute values. Distance traveled indicates the number of kilometers driven within the last 3 months. Kruskal–Wallis (*H*) and Chi-squared (*X*^*2*^*)* tests were performed to test the difference between group

### Driving scenario

#### Driving simulator

**Fig. 1:**
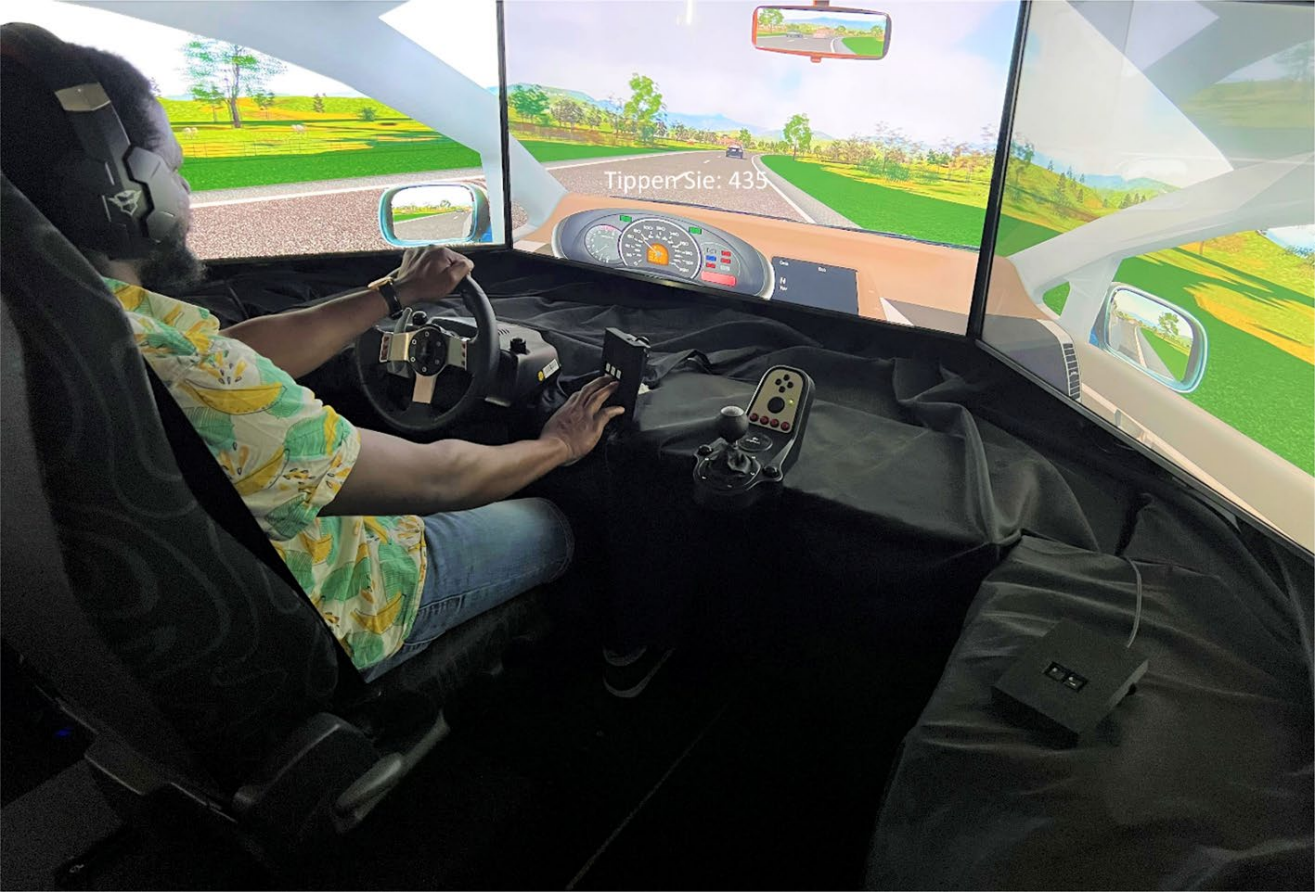
Driving environment. *Note.* The picture shows the driving simulator environment with a visual typing task displayed centrally on the middle monitor. The lead vehicle is visible ahead, while the rear vehicle is visible in the rear-view mirror

The driving simulator was set up with a VW Golf vehicle seat positioned in front of three 48″ monitors, mounted at eye level and offering a horizontal field of view of 195° (cf. Figure 1). A modified Logitech G27 steering wheel (Logitech International S.A., Lausanne, Switzerland) was fixed slightly to the left in front of the middle monitor for steering. The displayed graphics were rendered by commercially available driving software (Carnetsoft version 8.0, Groningen, The Netherlands). The graphics depicted a typical landscape with a winding rural road but no intersections or traffic lights. The landscape included clouds in a blue sky, mountains, little animal enclosures, grasslands, trees, traffic signs, gas stations, construction sites and vehicles that traveled at a constant speed on the oncoming traffic. Gas and brake pedals were used for acceleration and braking the car, respectively. The participants’ car was positioned between two other cars. The rear car was programmed to follow the participant’s car at a reasonable distance while the lead car drove at 70 km/h (~ 19.4 m/s). Participants were instructed to maintain a constant speed of 70 km/h, which was displayed on a simulated dashboard. They wore a regular headset with microphone for the presentation of auditory tasks and driving noise and for the recording of verbal data (ARG tasks). The verbal data were recorded continuously at 48,000 Hz and saved as "a .*wave file" on a separate computer using a free audio editor and recording application software (Audacity version, 3.3). Additionally, a conventional numeric keypad (used for TYPE tasks) was mounted to the right of the steering wheel within easy reach of participants. All keys except the numbers 1 to 6 were covered by black tape. Each driving trip was approximately 25 min.

#### Additional tasks

Two additional tasks were presented either aud or vis. The additional tasks were presented at fixed waypoints and irregular intervals, on average every 403.11 m (± 58.69), that is 69.93 km/h. SOA, that is the time window between the onset of the preceding task and the onset of the new task was on average about 21 s (Stojan & Voelcker-Rehage, 2021; Stojan et al., 2021; Wechsler et al., 2018). The additional tasks were modeled after natural activities, involving different sensory modalities, which required different types of cognitive functions and motor responses.

**TYPE**_**aud**_**.** Participants were asked to type a three-digit number with their right hand into the numeric keypad as quickly and as accurately as possible. Tasks were presented over the headphones and lasted for about 3 s.

**TYPE**_**vis**_**.** The TYPE_vis_ task was the same as the **TYPE**_**aud**_ except that the three-digit number was displayed for 5 s centrally on the middle monitor (font size = 30, color = white).

**ARG**_**aud**_**.** Participants were asked to provide a verbal argument for or against an issue of general interest** (**e.g., to state an argument “for” or “against” the use of electric vehicles) that was presented over headphones for about 3–4 s. Responses to all issues required more than a simple “yes” or “no”.

**ARG**_**vis**_**.** The ARG_vis_ task was the same as ARG_aud_ except that the task was presented centrally on the middle screen for 5 s (same font size and color as TYPE_vis)_.

**Fig. 2:**
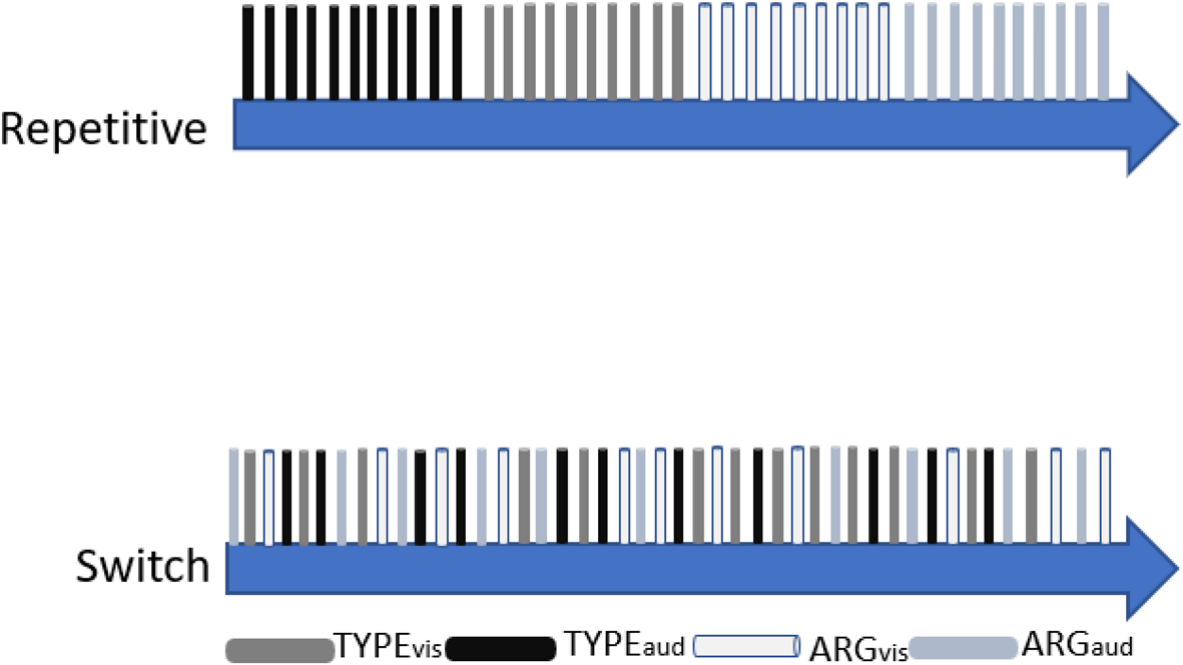
Overview of task presentation. *Note. *Serial order of task presentation in the repetitive and in the switch condition. Blue color refers to the driving task, and each of the bars depicted in black to light gray refers to one of the additional tasks. Abbreviations. Typing visual (TYPE_vis_); typing auditory (TYPE_aud_); argument visual (ARG_vis_); argument auditory (ARG_aud_). Driving conditions (repetitive or switch) were performed on different days and were counterbalanced for all participants

Additional tasks were presented in two conditions, repetitive and switch, which were administered in separate driving sessions, each of which included *n* = 60 additional tasks (cf. Figure 2). In the repetitive condition, each of the tasks explained above was presented repetitively 15 times (i.e., 15 trials per block). In the switch condition, all tasks were presented in an intermixed order. This means that the repetitive condition required dual-tasking within blocks and task-switching between blocks, while the switch condition required dual-tasking and task-switching on every trial. The concrete three-digit numbers and argumentation issues differed between trials; however, they were the same for all participants. It is important to note that no task was presented twice in a row in the switch condition. And correctness of responses was measured for both TYPE and ARG. Responses from TYPE were recorded by the driving simulator. Responses to ARG were protocolled and checked for validity by the researcher.

### Procedure

Each participant visited the laboratory twice, with one day of rest between sessions. On the first day, participants were given information about the experiment and signed a written consent form before beginning the experiment. Further, participants were instructed on how to perform the tasks. They were instructed to give the additional tasks the same priority as the driving task. Afterward, participants practiced the driving task together with the additional tasks for 4–5 min. Then, they began the main trials. The driving course for the practice trials was the same as that for the main trials. At the end of the experiment on the first day, they were given a demographic questionnaire to be filled out and brought back on the second day of testing.

On the second day, participants began the experiment without a practice trial; however, they were reminded how to perform the tasks. The order of driving conditions was counterbalanced for all participants. The order of task blocks in the repetitive condition was balanced using a Latin square design (i.e., in a given period of time, each task occurs once) for all participants. The order of task presentation in the switch condition was randomized but consistent for all participants. Participants also underwent cognitive and neurophysiological (fNIRS) testing; however, this study focuses on driving behavior performance. The results from the other tests will be communicated later.

### Data preprocessing

#### Driving behavior

Driving performance was analyzed for the time interval from 0 to 10 s after the onset of an additional task. This time interval was analyzed in our previous studies and showed sensitivity to additional tasks (Bock et al., 2021; Stojan et al., 2021). The driving parameters of main interest were average velocity (velocity, in m/s) and standard deviation of lateral position (SDLP, in m) of the participant’s vehicle. Velocity and SDLP indicate driving speed and ability to keep the vehicle centered in the lane, respectively. These outcome measures are commonly used in driving behavior research (Depestele et al., 2020). Further, we analyzed supplementary driving parameters; average distance to lead car, standard deviation of distance to lead car, standard deviation of driving velocity and average lateral position. This was to further elaborate our results (see supplementary materials, Appendix B for additional driving parameters). Outliers were excluded using the ± 3.29 SD criterion (Tabachnick & Fidell, 2007), separately for each participant, modality and task. The data were then averaged per task type (ARG and TYPE) and task modality (aud and vis) resulting in four averaged values per outcome parameter of interest (ARG_aud_, ARG_vis_, TYPE_aud_, TYPE_vis_, cf. below).

#### Additional tasks

The first trial of every block in the repetitive condition was excluded from the analysis because it involved a switch from one distinct additional task to another. To maintain an equal number of trials in the switch condition, corresponding trials in the switch condition were also excluded. Outliers were excluded using the ± 3.29 SD criterion (Tabachnick & Fidell, 2007), separately for each participant, modality and task. The data were then averaged per task type (ARG and TYPE) and task modality (visual and auditory) resulting in four averaged values. The following preprocessing steps were performed individually for TYPE and ARG:

**Typing (TYPE).** The RT of the typing task was determined as the time interval from stimulus onset until the first number was recorded for only correct trials. Typing accuracy was calculated as the percentage (%) of correct responses relative to the total number of presented tasks. Participants were excluded if their accuracy of responses was less than 50% per condition.

**Argumentation (ARG)**. First, the verbal data were down-sampled to 1000 Hz. Then, we manually checked every dataset to remove sounds that were not part of the verbal response, such as murmuring or giggling. Further, a moving average filter was applied to remove high-frequency noise from the signal. Similarly, as for the TYPE task, RT was then calculated as the interval between task presentation and the onset of a verbal response within a maximum response interval of 0–10 s (responses beyond 10 s were marked as misses). The RT ARG was determined by a self-developed R script (R Core Team, 2013). Argumentation accuracy (in %) was calculated as the percentage of all valid responses relative to the total number of presented argumentation tasks, separately for ARG_aud_ and ARG_vis_. The verbal responses were rated by one person blinded to the experimental condition as accurate (i.e., plausible responses to presented issues) or inaccurate. Similarly, as the TYPE task, participants were excluded if their accuracy of responses was less than 50% per condition.

In this study, only the RT for both ARG and TYPE tasks was submitted for analysis because accuracy levels were consistently close to 100% on all trials across all participants (cf. Supplementary data, Appendix A). Additionally, we focused on the performance differences between dual-tasking only and dual-tasking under switching conditions.

### Statistical analysis

The data were analyzed using RStudio version 4.3.1 (R Core Team, 2013). The “lme4” package was used for fitting linear mixed-effect models (LMMs) (Bates et al., 2014). LMMs were used to analyze the effect of condition (repetitive, switch) on the main driving parameters average velocity, SDLP, and on RT of additional tasks with condition as a fixed effect. The repetitive condition was set as the reference condition. Task (TYPE, ARG), Modality (aud, vis), Age, and Sex (males, females) were defined as covariates and added as fixed effects to control for their potential influences on the dependent variables, average velocity, SDLP, and RT. Additionally, random intercepts for participants and random slopes for condition were introduced to account for the inherent variability across individual participants and the potential variability associated with different conditions. Inclusion of covariates was strictly based on p-value, i.e., if *p* was ≥ 0.05, covariates were backwards stepwise excluded from the model.

Further, we conducted additional analysis to explore the specific effects of task and modality and their interaction with condition on the dependent variables. Therefore, we included a three-way interaction of Condition*Task*Modality in our model to determine how task and modality moderated the effect of condition on the dependent variables. Condition, Task, and Modality were used as main effects, and Age and Sex were entered as covariates. As we were only interested in determining whether the difference between conditions varied according to the specific task and its modality, we excluded the two-way interaction of Task*Modality from the model. For nonsignificant three-way interactions (i.e., *p* ≥ 0.05), we reran the LMM without the three-way interaction term to improve parsimony; however, we retained nonsignificant two-way interactions in the model for future research directions. Significant interactions with conditions were followed up with planned Tukey's HSD post hoc tests, so that the effect of condition was assessed separately for task type or modality.

We further explored whether the difference between conditions emerged for driving parameters other than our main parameters, average velocity and SDLP (cf. Supplementary data, Appendix B).

All models were fitted using maximum likelihood (ML) estimation. Confidence intervals (CIs) and generalized eta-squared (ηG^2^) were provided to interpret effects. Normal distribution of residuals was assessed using Kolmogorov–Smirnoff test. All dependent variables were normally distributed. Variance inflation factors (VIF) were examined for fixed effects and covariates to detect multicollinearity between the predictors and the dependent variables. We interpreted ηG^2^ as follows: < 0.06 = small, 0.06–0.14 = medium and > 0.14 = large. All *p*-values < 0.05 were interpreted as significant.

## Results

**Fig. 3:**
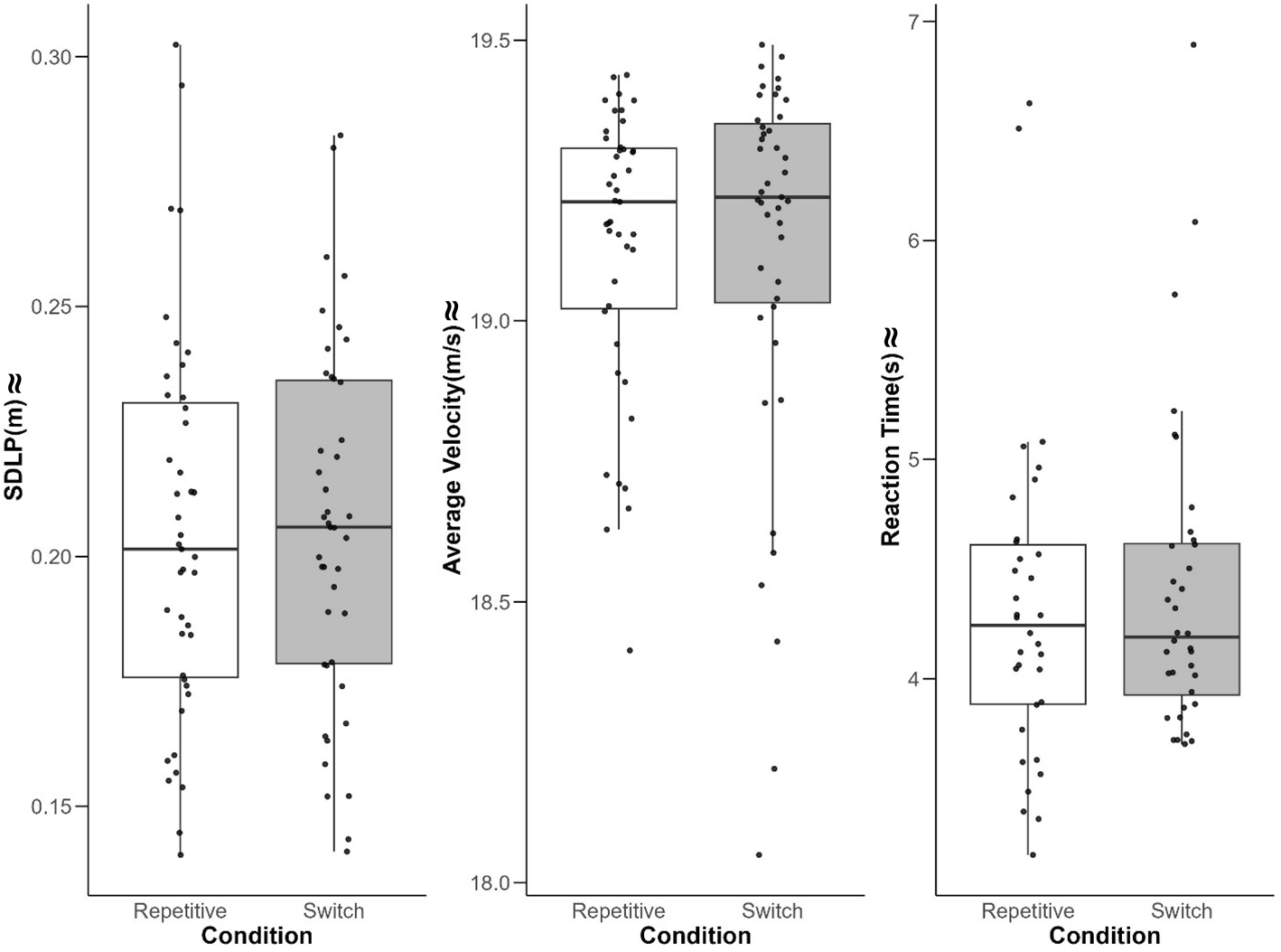
Results for the main performance parameters separated by condition. *Note.* Distribution of behavioral performance with individual data points for the three main performance parameters: SDLP (standard deviation of lateral position), average velocity and reaction time of additional task for the conditions repetitive and switch

**Main analysis on driving parameters.** The LMM results for SDLP (cf. Table 2) indicated no main effect for Condition (*t* (42) = 0.30, *p* = 0.77, CI: [ − 0.01–0.01], ηG^2^ = 0.00)*.* This implies that task presentation (repetitive vs. switch) did not affect participants’ SDLP during driving while performing additional tasks (cf. Figure 3). Likewise, for average velocity, LMM (cf. Table 2) revealed no main effect for Condition (*t (*42) = − 0.22, *p* = 0.83, CI: [ − 0.09 – 0.07], ηG^2^ = 0.00)*.* We thus found no evidence for task presentation to affect participants’ mean velocity during additional task performance while driving (cf. Figure 3).

**Table 2:** LMM results with the driving parameters SDLP and average velocity as dependent variables and condition as independent variable

Predictors	Coefficient	Std.error	*t* value	*P*	95% CI Lower	95% CI Upper	ηG^2^
*SDLP (included terms)*							
Intercept	0.18	0.01	27.49	< 0.001***	0.17	0.19	
Condition (Switch)	0.00	0.00	0.30	0.77	-0.01	0.01	< 0.01
Task (TYPE)	0.01	0.00	4.34	< 0.001***	0.01	0.02	< 0.01
Modality (Vis)	0.01	0.00	2.89	<0 .01**	0.00	0.01	< 0.01
*Excluded terms*							
Sex (M)	− 0.01	0.01	− 0.59	0.56	− 0.03	0.01	0.01
Age	0.00	0.00	− 1.01	0.32	− 0.01	0.00	0.02
*Average velocity (included terms)*							
Intercept	19.14	0.04	497.88	<0 .001***	19.06	19.21	
Condition (Switch)	− 0.01	0.04	− 0.22	0.83	− 0.09	0.07	< 0.01
*Excluded terms*							
Task (TYPE)	0.00	0.02	0.40	0.69	− 0.03	0.04	< 0.01
Modality (Vis)	0.03	0.02	1.29	0.20	− 0.01	0.06	< 0.01
Sex (M)	− 0.04	0.08	− 0.49	0.63	− 0.20	0.13	< 0.01
Age	0.01	0.02	0.93	0.36	− 0.02	0.04	0.02
*Reaction time (included terms)*							
Intercept	6.18	0.11	55.85	< 0.001***	5.96	6.40	
Condition (Switch)	0.08	0.07	1.21	0.24	− 0.05	0.21	0.04
Task (TYPE)	− 2.83	0.03	− 88.09	< 0.001***	− 2.90	− 2.77	0.67
Modality (Vis)	− 0.41	0.03	− 12.89	< 0.001***	− 0.47	− 0.35	0.04
*Excluded terms*							
Age	0.01	0.04	0.35	0.73	− 0.06	0.08	< 0.01
Sex(M)	0.20	0.18	1.11	0.28	− 0.18	0.58	0.03

Condition included two levels (repetitive, switch), Task consisted of two levels (ARG, TYPE), and Modality consisted of two levels (Aud, Vis*). CI* = confidence interval. Task, modality, age, and sex were entered as covariates. Covariates were backward stepwise excluded from the model if *p* > .05 (excluded terms). ηG^2^ = general eta-squared. Included and excluded fixed effect terms are strictly based on *p*-values

***:* p*-value < .001, **:* p*-value < .01, *:* p*-value <. 05

**Main analysis on RT of additional tasks.** For RT, LMM (cf. Table 2), revealed no main effect for condition (*t* (32.65) = 1.21, *p* = 0.24, CI: [ − 0.05–0.21], ηG^2^** = **0.04) on RT. Similar to the driving parameters, there was no evidence for task presentation to affect participants’ ability to perform additional tasks while driving (cf. Figure 3).

**Fig. 4:**
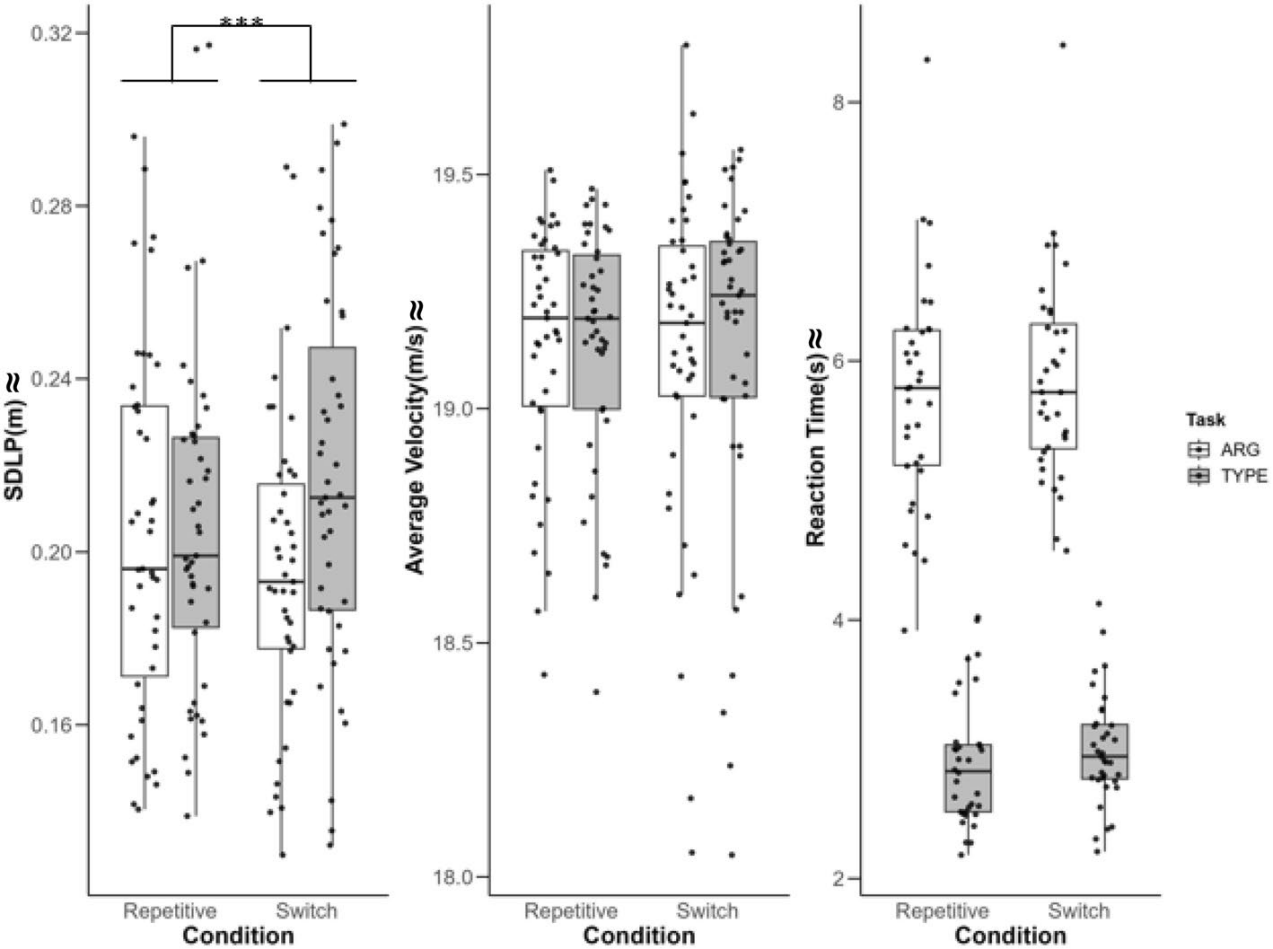
Results of the main performance parameters (additional analysis) separated by condition and task type. *Note.* Distribution of behavioral performance for each condition per task with individual data points for the three main performance parameters SDLP (standard deviation of lateral position), average velocity and reaction time of additional task for the conditions repetitive and switch, separated by task type*.* Asteriks indicate significant interaction effect

**Fig. 5:**
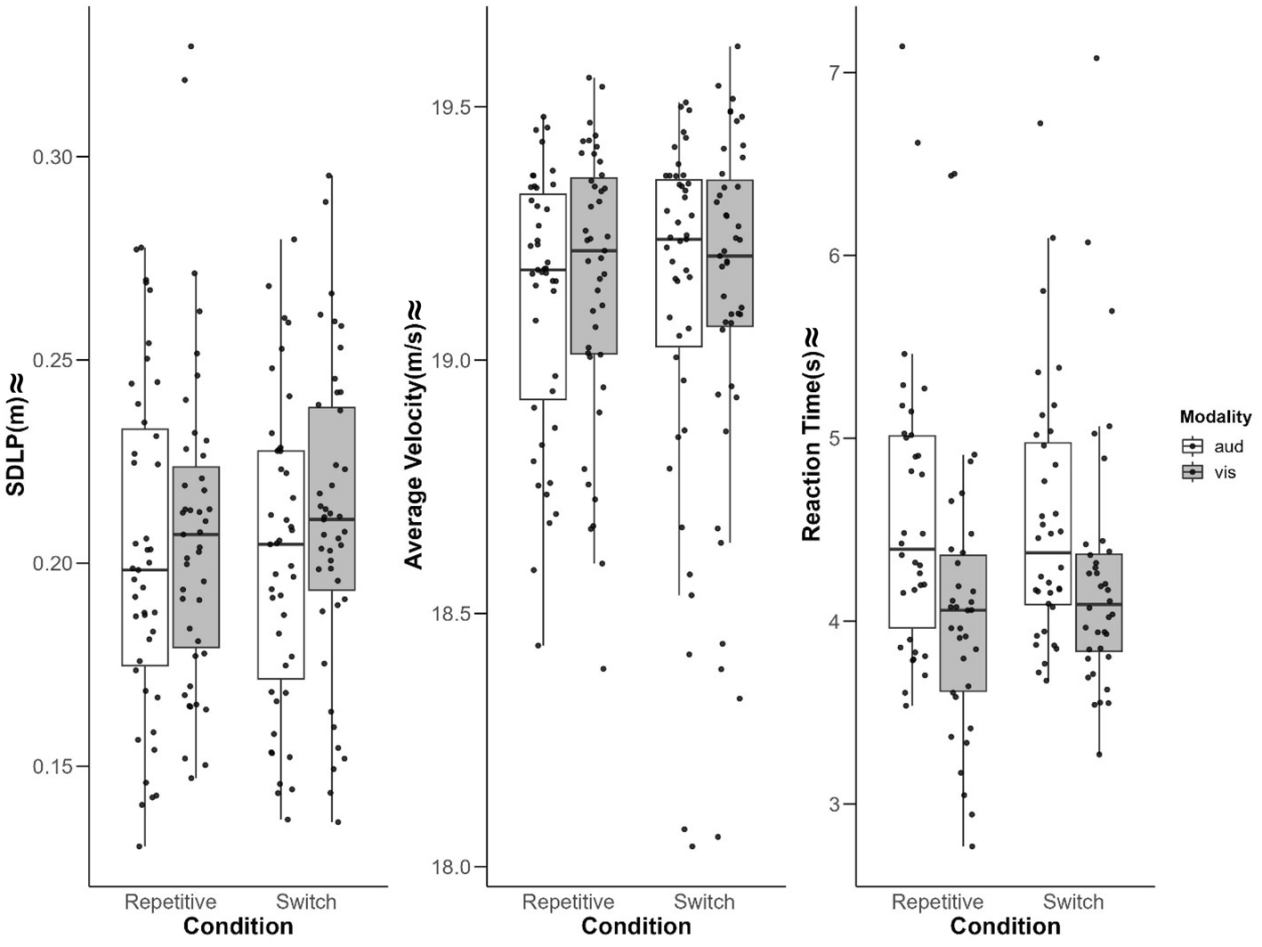
Results of the main performance parameters (additional analysis) separated by condition and Modality. *Note.* Distribution of behavioral performance for each condition per task modality with individual data points for the three main performance parameters SDLP (standard deviation of lateral position), average velocity and reaction time of additional task* for the conditions repetitive and switch and separated by modality*

**Additional analysis on driving parameters**. When adding the three-way interaction of Condition*Task*Modality to our model, the LMM results for SDLP (cf. Table 3) indicated a main effect for Condition (*t* (162.20) = − 2.18, *p* = 0.03, *CI*: [ − 0.02 – 0.00], *ηG*^2^ < 0.01), with participants showing a stronger variability in the switch condition (*M* = 0.21, *SE* = 0.01) than the repetitive condition (*M* = 0.20, *SE* = 0.01). Further, there was a significant Condition*Task interaction (*t* (5070) = 4.33, *p* < 0.001, *CI*: [0.01–0.03], *ηG*^*2*^ < 0.01)*.* The planned Turkey’s HSD post hoc test showed a significant difference (β = 0.01, *p* = 0.02, *ηG*^*2*^ = *0.01*) between the conditions during the TYPE task, i.e., a higher variability in the switch condition (*M* = 3.02, *SE* = 0.09) than the repetitive condition (*M* = 2.95, *SE* = 0.11), but not during the ARG task (β = 0.01, p = 0.06, *ηG*^*2*^ = 0.01; repetitive condition: *M* = 5.87, *SE* = 0.09, switch condition: *M* = 5.77, *SE* = 0.11). Finally, we found no significant Condition*Modality interaction (*t* (5070) = 1.23, *p* = *0.2*1, *CI*: [ − 0.01–0.02], *ηG*^*2*^ < 0.01) (cf. Figures 4 and 5).

**Table 3:** LMM results from the additional analysis adding the Condition*Task and Condition*Modality interactions

Predictors	Coefficient	Std.Error	*t* value	*P*	95% CI Lower	95% CI Upper	*ηG*^*2*^
*Standard deviation of lateral position, SDLP (included terms)*							
Intercept	0.20	0.01	31.41	< 0.001***	0.19	0.21	
Condition (switch)	− 0.01	0.01	− 2.18	0.03*	− 0.02	0.00	< *0.01*
Task (TYPE)	0.00	0.00	− 0.13	0.90	− 0.01	0.01	< *0.01*
Modality (Vis)	0.00	0.00	0.82	0.41	0.00	0.01	< *0.01*
Condition*Task	0.02	0.01	4.33	< 0.001***	0.01	0.03	< *0.01*
Condition*Modality	0.01	0.01	1.26	0.21	− 0.01	0.02	< *0.01*
*Excluded terms*							
Sex (M)	− 0.01	0.01	− 1.08	0.29	− 0.03	0.01	*0.01*
Age	0.00	0.00	− 1.01	0.32	0.00	0.00	*0.02*
Condition*Task* Modality	0.01	0.01	0.76	0.44	− 0.01	0.03	< *0.01*
*Average velocity (included terms)*							
Intercept	19.12	0.43	443.23	< 0.001***	18.02	19.47	
Condition (Switch)	− 0.01	0.05	− 0.11	0.92	− 0.10	0.10	< *0.01*
Task (TYPE)	0.00	0.03	− 0.12	0.91	− 0.06	0.05	< *0.01*
Modality (Vis)	0.04	0.03	1.37	0.17	− 0.01	0.10	< *0.01*
Condition*Task	0.02	0.04	0.47	0.64	− 0.06	0.09	< *0.01*
Condition*Modality	− 0.03	0.04	− 0.64	0.52	− 0.11	0.04	< *0.01*
*Excluded terms*							
Sex (M)	− 0.03	0.08	− 0.49	0.63	− 0.20	0.13	*0.01*
Age	0.01	0.02	0.93	0.36	− 0.02	0.04	*0.02*
Condition*Task* Modality	0.12	0.08	1.58	0.11	− 0.02	0.28	< *0.01*
*Reaction time, RT, in additional tasks (included terms)*							
Intercept	6.01	0.11	54.68	< 0.001***	5.79	6.23	
Condition (Switch)	0.03	0.08	0.41	0.69	− 0.12	0.20	*0.04*
Task (TYPE)	− 2.82	0.05	− 61.35	< 0.001***	− 2.91	− 2.73	*0.67*
Modality (Vis)	− 0.48	0.05	− 10.47	<0 .001***	− 0.57	− 0.39	*0.04*
Condition*Task	− 0.03	0.06	− 0.46	0.64	− 0.16	*0.10*	< *0.01*
Condition*Modality	0.13	0.06	1.20	0.05	0.00	*0.25*	< *0.01*
*Excluded terms*							
Sex (M)	0.21	0.18	1.08	0.29	− 0.20	0.57	*0.03*
Age	0.01	0.04	0.38	0.70	− 0.06	0.08	< *0.01*
Condition*Task* Modality	− 0.06	0.13	− 0.45	0.65	− 0.31	0.19	< *0.01*

Condition included two levels (repetitive, switch), Task consisted of two levels (ARG, TYPE) and Modality consisted of two levels (Aud, Vis*). CI* = confidence interval. Age and Sex were entered as covariates. Covariates were backward stepwise excluded from the model if *p* ≥ .05 (excluded terms). ηG^2^ = general eta-squared

***:* p*-value < .001, **:* p*-value < .01, *:* p*-value <. 05

For average velocity, however, LMM (cf. Table 3) revealed no main effect for condition (*t* (88.36) = − 0.11, *p* = 0.92, *CI*: [-0.10 – 0.10], *ηG*^2^ < 0.01) and no significant Condition*Task (*t* (5070) = 0.47, *p* = 0.64, *CI*: [ − 0.06 – 0.09], *ηG*^2^ < 0.01), and Condition*Modality interaction (*t* (5070) = − 0.64, *p* = 0.52, *CI*: [ − 0.11 – 0.04], *ηG*^2^ < 0.01), suggesting that the task type and modality did not affect participants’ mean velocity during additional task performance while driving (cf. Figures 4 and 5).

**Additional analysis for RT of additional tasks.** The performance of the additional tasks (RT), LMM (cf. Table 3), revealed no main effect for Condition (*t* (70.49) = 0.65, *p* = 0.69, *CI*: [ − 0.12–0.20], *ηG*^2^ = 0.04), no Condition*Task interaction (*t* (3794.95) = − 0.46, *p* = 0.64, *CI*: [ − 0.16 – 0.10], *ηG*^2^ < 0.01), and no Condition*Modality interaction (*t* (3771.77) = 1.99, *p* = 0.05, *CI*: [0.00 – 0.25], *ηG*^2^ < 0.01)*.* This again suggests that the different conditions (repetitive vs. switch) did not impact on the participants’ ability to perform additional tasks while driving (cf. Figures 4 and 5).

Since a significant effect of condition and Condition*Task was only found for SDLP after including the interaction effect terms, we extended our analyses to the following supplementary driving parameters: average distance to lead car, standard deviation of distance to lead car, standard deviation of driving velocity, and average lateral position to test the sensitivity of our findings. However, the effect of condition was not significant for any supplementary parameter, neither in the main analysis nor in the additional analysis (cf. Supplementary data, Appendix B). However, we found a significant Condition*Task and Condition * Modality interactions only for average distance which, however, were not substantiated by post hoc tests (cf. Supplementary data, Appendix C).

## Discussion

The detrimental effects of performing two or more tasks either simultaneously (i.e., dual-tasking) or sequentially (i.e., task-switching) have previously been investigated separately in multitasking research. In contrast, the present study aimed to investigate the additional effects of task-switching on dual-task performance. We conducted this investigation in a more naturalistic setting compared to classical laboratory experimental designs, using a driving scenario to enhance ecological validity while maintaining a high level of controllability. In our previous studies (Bock et al., 2021; Stojan et al., 2024), we observed multitasking deficits when driving was combined with a mix of additional tasks (dual-tasking and task-switching). We now compared this condition with a setup in which additional tasks are presented block-wise (i.e., dual-task without task-switching) to eliminate effects of task-switching. Contrary to our hypothesis, performance seemed to be comparable between the repetition and switch conditions for all dependent variables, suggesting that there is no additional effect of task-switching on driving parameters or RT of additional tasks. However, when advancing our analysis for the type of additional task, we found better driving performance in the repetitive than switch condition for the typing task, but not for the argumentation task. Considering that this significant effect emerged only for one dependent variable, we conclude that the evidence in favor of our hypothesis is quite limited, but that the effect might be task dependent. In other words, switching between additional tasks seems to have only very limited impact on multitasking if compared to repeating a given additional task.

In our main analysis, we were not able to reveal a difference between dual-tasking without task-switching and dual-tasking with task-switching. The overall lack of substantial performance decrements in the task-switching condition compared to the repetitive condition in our study might be explained by a decay of the task-set and/or advanced reconfiguration due to task preparation effects (Kiesel et al., 2010). In task-switching paradigms, preparation effects refer to the processes that improve performance in the switch condition (Kiesel et al., 2010), specifically, the ability to prepare for the upcoming task before the switch occurs. It therefore is plausible that, in our study, the long SOA of approximately 21 s allowed the persistent task-set effect from the preceding task to decay, thus increasing the preparation time for the new task, potentially preventing any effect on the new additional task during continuous driving. Although there is evidence of substantial task-switching effects observed with SOAs as long as 6–12 s (Barber & Carter, 2005; Bock et al., 2021; Braver et al., 2003; Stojan et al., 2024) in driving contexts, it is conceivable that the persistent task-set effect dissipated before the onset of the new task at the extended SOA of 21 s, thereby eliminating the additive effect in the switch condition. Indeed, several studies (Allport & Wylie, 1999, 2000; Meiran, 1996; Rogers & Monsell, 1995) have demonstrated that a prolonged interval between the response in the preceding task and the onset of the next task stimulus enhances performance in the switch condition. This improvement is attributed to either advanced reconfiguration (Rogers & Monsell, 1995) for the upcoming task or decay of the preceding task-set (Allport & Wylie, 1999; Altmann & Gray, 2008). It, therefore, remains an open question whether persistent task-set effects indeed dissipate when SOAs exceed 12 s and approache 21 s, as observed in the present study.

An alternative interpretation for the lack of a substantial effect of task-switching in our study relates to the complexity of the driving task (Schuch et al., 2020). Controlling the lateral lane position and distance to the lead car by means of the steering wheel and two pedals, all this in the presence of realistic distractions moving by, may demand a substantial portion of the participants’ cognitive resources. Responding to additional tasks atop of that is likely to demand resources as well, with little cognitive resources remaining to form task-sets. If no task-sets are formed, the repetitive condition may offer no advantage over the switch condition, thus explaining the lack of significant differences between the condition in our study.

Further, we investigated whether the effects of condition on driving parameters and RT of additional tasks were moderated by the specific task demands, including the task type and its modality. While modality did not significantly moderate the effect of condition, we observed a moderation by task for variability in lane keeping (SDLP), but not for mean velocity or the RT of the additional task. Participants' ability to maintain the car's position in the lane was poorer in the switch condition compared to the repetitive condition, but this was only the case for the typing task. Previous dual-task studies demonstrated that typing task relies strongly on visual attention (Chaparro et al., 2005; Getzmann et al., 2018), while controlling the lateral position of the car also strongly depends on visuospatial attention (Karthaus et al., 2020; Stojan & Voelcker-Rehage, 2021). It is therefore plausible that the observed Condition * Task interaction reflects visuospatial competition between the driving task and the typing task, which is likely not the case for the argumentation task. However, when considering the effect of switching between different dual-task performances—specifically, switching from an argumentation task while driving to a typing task while driving on SDLP—resource competition may magnify the task-set effect. Mechanistically, this task-specific effect could reflect a persistent task-set effect from the argumentation task on the typing task. Such effects may be explained by differences in the duration of the information processing stage required for each task (Koch et al., 2018). Specifically, the processing interval, spanning from perceptual encoding to response execution, is likely shorter for a "three-digit number" task than for "stating an argument for or against the use of electric vehicles" (see Method section for task details). This suggests that the task-set for the argumentation tasks took longer to decay in working memory compared to typing tasks, making the reconfiguration from the argumentation task’s task-set to the typing task more demanding than the reverse. Consequently, in the switch condition, when an argumentation task precedes a typing task, residual effects from the argumentation task may interfere with the typing task, resulting in greater variability in the SDLP. In contrast, modality did not appear to further moderate the task-set effect. Taken together, we found limited evidence that task-specific effects might influence task-switching during dual-task performance.

Our study is the first to investigate the combination of task-switching and dual-tasking within a single setting in a realistic, everyday-like scenario. Compared to typical task-switching studies, our study used a scenario that is more naturalistic, making our experiment one step closer to the real world. This suggests that our findings may generalize to real life more readily compared to the findings from traditional laboratory experiments (Chaytor & Schmitter-Edgecombe, 2003). Furthermore, our study differs from classical task-switching research by using a condition that involves switches on all trials, rather than just half of the trials, as in the classical “mixed” condition. This distinction could potentially enhance switching performance by improving cognitive flexibility, as participants had to keep multiple task-sets in working memory and, therefore, improved their ability to react to new tasks (Dreisbach & Fröber, 2019). Future studies therefore should compare the switching performance with and without interspersed repeat trials, to better understand the dynamics of task-switching.

A limitation of our study is that participants were tested only in repetitive and switch conditions, without an additional single-task condition. Although our previous studies with a similar driving paradigm (Bock et al., 2021; Stojan & Voelcker-Rehage, 2021; Stojan et al., 2024) documented multitasking costs when comparing a switch condition to a single-task condition, we cannot be absolutely certain that the particular sample recruited for the present study would exhibit similar multitasking costs. However, such a difference between the present and previous samples is highly unlikely; it is theoretically conceivable and therefore worth mentioning. The reason for not including a single-task control in the present study was to avoid fatigue effects in our rather monotonous experiment. Additionally, the experimental design only allowed us to examine task type effects but not task difficulty. For example, typing a 3-digit number versus a 5-digit number differs in difficulty, yet it remains uncertain which of these would be more comparable to the complexity of the argumentation task. Investigating the effect of task difficulty on task-set effects would be an interesting direction for future studies. Furthermore, the two different additional tasks, typing and argumentation, with their differential responses (verbal or manual), may give rise to stimulus–response compatibility effects on both driving and additional task performances (Fintor et al., 2018; Stelzel & Schubert, 2011). However, systematically investigating stimulus–response compatibility is beyond the scope of this study. Future studies should consider including stimulus–response compatibility in their design to understand its impact on both driving and additional task performance. Finally, our study employed a SOA of 21 s, which is a considerably long SOA if compared to classical switching paradigms. We selected this long SOA as it is realistic for driving and as it is consistent with our earlier work (Bock et al., 2021; Stojan et al., 2024). However, it would be interesting in future research to study the interplay of dual-task and task-switching under a range of different SOA.

Overall, this study investigated the effects of task-switching on multitasking in an everyday-like scenario, simulated driving. We found only quite limited evidence for performance decrements when additional tasks were presented in a mixed rather than in a repetitive sequence, possibly because already the repetitive sequence precluded task preparation. We, however, found limited evidence that task type might influence such effect, calling for future studies carefully manipulating task type, switching design, and stimulus onset asynchrony.

## Generalization

We were interested in whether driving performance is similar while performing an additional task, regardless of whether the additional task is always the same or whether it differs (task-switching). Our results indicate that the order of the tasks does so only for visually distracting tasks. We expect our results to generalize to situations in which younger adults perform a driving task as long as the task characteristics are similar (e.g., visual distraction task). Whether these results generalize to other age groups, such as older drivers, needs to be investigated. The results should be considered in the design of driver assistance systems.

## Supplementary Information

Additional file1

## Data Availability

The authors will make the raw data supporting the conclusions of this manuscript available upon reasonable request.
