# Supplementary material for: Multitasking: does task-switching add to the effect of dual-tasking on everyday-like driving behavior?
Source: Cogn Res Princ Implic. 2025 Feb 8;10:5. doi: 10.1186/s41235-025-00611-y (PMC11807033; doi:10.1186/s41235-025-00611-y)
Supplement: Supplementary file 1 — Additional file1 [file 41235_2025_611_MOESM1_ESM.docx]

# **Appendix A**

# **Descriptive statistics**

**Table A1**

***Descriptive statistics for main performance parameters***

|  | **Repetitive** | | **Switch** | |
| --- | --- | --- | --- | --- |
|  | ***M*** | ***SD*** | ***M*** | ***SD*** |
| **Driving Performance** | | | | |
| **Standard deviation of lateral position (SDLP)** | | | | |
| SDLP (ARG) | 0.20 | 0.10 | 0.21 | 0.09 |
| SDLP (ARGaud) | 0.21 | 0.11 | 0.19 | 0.09 |
| SDLP(ARGvis) | 0.21 | 0.11 | 0.20 | 0.11 |
| SDLP (TYPE) | 0.21 | 0.10 | 0.22 | 0.11 |
| SDLP (TYPEaud) | 0.21 | 0.10 | 0.21 | 0.10 |
| SDLP (TYPEvis) | 0.21 | 0.10 | 0.22 | 0.11 |
| Average velocity | | | | |
| Velocity (ARG) | 19.14 | 0.70 | 19.12 | 0.78 |
| Velocity (ARGaud) | 19.10 | 0.69 | 19.13 | 0.79 |
| Velocity (ARGvis) | 19.17 | 0.70 | 19.11 | 0.78 |
| Velocity (TYPE) | 19.13 | 0.71 | 19.14 | 0.84 |
| Velocity (TYPEaud) | 19.13 | 0.73 | 19.11 | 0.91 |
| Velocity (TYPEvis) | 19.14 | 0.68 | 19.16 | 0.76 |
| **Additional Task Performance** | | | | |
| **Reaction Time (RT)** | | | | |
| RT (ARG) | 5.75 | 1.50 | 5.85 | 1.47 |
| RT (ARGaud) | 5.96 | 1.36 | 6.01 | 1.41 |
| RT (ARGvis) | 5.54 | 1.58 | 5.69 | 1.52 |
| RT (TYPE) | 2.87 | 0.70 | 2.99 | 0.68 |
| RT (TYPEaud) | 3.11 | 0.74 | 3.18 | 0.73 |
| RT (TYPEvis) | 2.65 | 0.58 | 2.79 | 0.57 |
| **Accuracy** |  |  |  |  |
| RT (ARG) | 98.57 | 6.05 | 97.58 | 5.74 |
| RT (ARGaud) | 98.77 | 5.63 | 98.01 | 6.14 |
| RT (ARGvis) | 97.65 | 7.85 | 97.96 | 7.25 |
| RT (TYPE) | 98.89 | 6.07 | 97.39 | 6.86 |
| RT (TYPEaud) | 98.59 | 6.47 | 98.81 | 5.37 |
| RT (TYPEvis) | 98.56 | 7.85 | 97.97 | 4.75 |

*Note.* Means (M) and standard deviation are (SD) presented for the main performance parameters; Standard deviation of lateral position, Average velocity and Reaction of additional tasks. Abbreviations: ARG= Argument, TYPE= Typing, aud= Auditory, vis= Visual, RT= Reaction time.

# **Appendix B**

# **Findings from the primary and exploratory analysis for supplementary driving parameters**

**Table B1**

**LMM results from the primary analysis (supplementary driving parameters)**

| **Predictors** | **Coefficient** | **Std.Error** | **t value** | ***P*** | **95% CI Lower** | **95% CI Upper** | **ηG²** |
| --- | --- | --- | --- | --- | --- | --- | --- |
| **Average distance (Included Terms)** | | | | | | | |
| Intercept | ***60.83*** | 2.66 | 22.89 | **<.001***** | 55.56 | 66.09 |  |
| Condition (Switch) | -2.21 | 3.289 | -0.67 | .51 | -8.72 | 4.30 | 0.01 |
| Task (TYPE) | 2.02 | 0.47 | 4.25 | **<.001***** | 1.09 | 2.94 | <0.01 |
| **Excluded Terms** | | | | | | | |
| Modality (Vis) | 0.63 | 0.47 | 1.33 | .19 | -0.30 | 1.56 | <0.01 |
| Sex (M) | 7.31 | 4.21 | 1.74 | .09 | -0.98 | 15.60 | 0.07 |
| Age | 0.05 | 0.83 | 0.06 | .95 | -1.60 | 1.70 | <0.01 |
| **SD average distance (Included Terms)** | | | | | | | |
| Intercept | 1.90 | 0.08 | 23.77 | **<.001***** | 1.75 | 2.06 |  |
| Condition (Switch) | -0.05 | 0.09 | -0.59 | .56 | -0.23 | 0.12 | <0.01 |
| **Excluded Terms** | | | | | | | |
| Task (TYPE) | 0.03 | 0.04 | 0.75 | .45 | -0.05 | 0.11 | <0.01 |
| Modality (Vis) | 0.00 | 0.04 | -0.10 | .92 | -0.08 | 0.08 | <0.01 |
| Sex (M) | 0.00 | 0.01 | 0.03 | .98 | -0.26 | 0.27 | <0.01 |
| Age | 0.00 | 0.00 | -0.26 | .79 | -0.05 | 0.045 | <0.01 |
| **Average lateral position (Included Terms)** | | | | | | | |
| Intercept | -0.04 | 0.00 | -14.19 | **<.001***** | -0.46 | -0.35 |  |
| Condition (Switch) | 0.00 | 0.00 | -0.18 | .86 | -0.04 | 0.03 | <0.01 |
| Modality (Vis) | 0.00 | 0.01 | 3.97 | **0.00**** | 0.01 | 0.03 | <0.01 |
| **Excluded Terms** | | | | | | | |
| Age | 0.01 | 0.00 | 0.61 | .54 | -0.01 | 0.02 | <0.01 |
| Sex(M) | 0.01 | 0.01 | 1.69 | .10 | -0.01 | 0.19 | 0.07 |
| Task (TYPE) | 0.00 | 0.01 | -0.21 | .84 | -0.01 | 0.01 | <0.01 |
| **SD Velocity (Included Terms)** | | | | | | | |
| Intercept | 0.03 | 0.00 | 11.34 | **<.001***** | 0.25 | 0.35 |  |
| Condition (Switch) | 0.00 | 0.00 | 1.46 | .15 | -0.01 | 0.07 | <0.01 |
| Task (TYPE) | 0.01 | 0.01 | 2.10 | **.04*** | -0.04 | 0.00 | <0.01 |
| Modality (Vis) | 0.00 | 0.01 | -2.33 | **.02*** | 0.01 | 0.03 | <0.01 |
| **Excluded Terms** |  |  |  |  |  |  |  |
| Age | 0.01 | 0.00 | -0.50 | .62 | -0.03 | 0.02 | <0.01 |
| Sex(M) | 0.01 | 0.01 | -1.26 | .21 | -0.17 | 0.04 | 0.04 |

*Note.* Condition included two levels (Repetitive, Switch), Task consisted of two levels (ARG, TYP) and Modality consisted of two levels (Aud, Vis*).* CI=confidence interval. Task, Modality, Age and Sex were entered as covariates. Covariates were backward stepwise excluded from the model. ηG² = general eta-squared

**Table B2**

***LMM results from exploratory analysis (supplementary driving parameters)***

| **Predictors** | **Coefficient** | **Std.Error** | **t value** | ***P*** | **95% CI Lower** | **95% CI Upper** | **ηG²** |
| --- | --- | --- | --- | --- | --- | --- | --- |
| **Average distance (included Terms)** | | | | | | | |
| Intercept | 57.77 | 2.69 | 21.49 | **<.001***** | 52.45 | 63.09 |  |
| Condition (switch) | 3.27 | 3.35 | 0.97 | .34 | -3.37 | 9.90 | 0.01 |
| Task (TYPE) | 5.32 | 0.67 | 7.99 | **<.001***** | 4.02 | 6.63 | <0.01 |
| Modality (Vis) | 2.79 | 0.67 | 4.20 | **<.001***** | 1.49 | 4.10 | <0.01 |
| Condition*Task | -6.62 | 0.94 | -7.03 | **<.001***** | -8.46 | -4.77 | <0.01 |
| Condition*Modality | -4.33 | 0.94 | -4.60 | **<.001***** | -6.18 | -2.49 | <0.01 |
| **Excluded Terms** |  |  |  |  |  |  |  |
| Sex (M) | 7.33 | 4.15 | 1.77 | **.**09 | -0.96 | 15.61 | 0.07 |
| Age | -0.03 | 0.82 | -0.04 | .97 | -1.64 | 1.58 | <0.01 |
| Condition*Task* Modality | 0.71 | 1.88 | 0.38 | .70 | -2.97 | 4.39 | <0.01 |
| **SD Average distance (Included Terms)** | | | | | | | |
| Intercept | 1.89 | 0.09 | 22.19 | **<.001***** | 1.72 | 2.05 |  |
| Condition (Switch) | -0.02 | 0.11 | -0.19 | .85 | -0.23 | 0.19 | 0.04 |
| Task (TYPE) | 0.04 | 0.06 | 0.69 | .49 | -0.07 | 0.15 | 0.67 |
| Modality (Vis) | 0.02 | 0.06 | 0.33 | .74 | -0.09 | 0.13 | 0.04 |
| Condition*Task | -0.02 | 0.08 | -0.22 | .83 | -0.18 | 0.14 | <0.01 |
| Condition*Modality | -0.05 | 0.08 | -0.57 | .57 | -0.21 | 0.11 | <0.01 |
| **Excluded Terms** |  |  |  |  |  |  |  |
| Sex (M) | 0.00 | 0.01 | 0.03 | .98 | -0.27 | 0.27 | <0.01 |
| Age | 0.00 | 0.01 | 0.03 | .98 | -0.06 | 0.05 | <0.01 |
| Condition*Task* Modality | -0.03 | 0.02 | -1.89 | .06 | -0.63 | 0.01 | <0.01 |
| **Average lateral position (Included Terms)** | | | | | | | |
| Intercept | -0.41 | 0.03 | -13.87 | **<.001***** | -0.45 | -0.34 |  |
| Condition (Switch) | -0.01 | 0.02 | -0.45 | .65 | -0.04 | 0.03 | <0.01 |
| Task (TYPE) | 0.00 | 0.01 | -0.34 | .73 | -0.02 | 0.01 | <0.01 |
| Modality (Vis) | 0.02 | 0.01 | 2.34 | **.02*** | 0.00 | 0.03 | <0.01 |
| Condition*Task | 0.00 | 0.01 | 0.28 | .78 | -0.02 | 0.03 | <0.01 |
| Condition*Modality | 0.01 | 0.01 | 0.66 | .51 | -0.01 | 0.03 | <0.01 |
| **Excluded Terms** |  |  |  |  |  |  |  |
| Sex (M) | 0.09 | 0.05 | 1.75 | .09 | -0.01 | 0.19 | 0.07 |
| Age | 0.01 | 0.01 | 0.48 | .64 | -0.01 | 0.02 | <0.01 |
| Condition*Task* Modality | 0.04 | 0.02 | 1.80 | .07 | 0.00 | 0.09 | <0.01 |
| **SD velocity (Included Terms)** | | | | | | | |
| Intercept | 0.30 | 0.03 | 11.20 | **<.001***** | 18.02 | 19.47 |  |
| Condition (Switch) | 0.02 | 0.02 | 0.91 | .36 | -0.10 | 0.10 | <0.01 |
| Task (TYPE) | 0.02 | 0.01 | 1.35 | ***.18*** | -0.06 | 0.05 | <0.01 |
| Modality (Vis) | -0.03 | 0.02 | -2.20 | **.03*** | -0.01 | 0.10 | <0.01 |
| Condition*Task | 0.00 | 0.02 | 0.20 | .84 | -0.06 | 0.09 | <0.01 |
| Condition*Modality | 0.01 | 0.02 | 0.78 | .44 | -0.11 | 0.04 | <0.01 |
| **Excluded Terms** |  |  |  |  |  |  |  |
| Sex (M) | -0.06 | 0.05 | -1.26 | .21 | -0.16 | 0.04 | 0.04 |
| Age | 0.01 | 0.02 | -0.50 | .62 | -0.03 | 0.02 | <0.01 |
| Condition*Task* Modality | -0.05 | 0.04 | -1.59 | .11 | -0.12 | 0.01 | <0.01 |

*Note.* Condition included two levels (Repetitive, Switch), Task consisted of two levels (ARG, TYP) and Modality consisted of two levels (Aud, Vis*).* CI=confidence interval. Task, Modality, Age and Sex were entered as covariates. Covariates were backward stepwise excluded from the model. ηG² = general eta-squared

**Figure B1**

*Average distance to lead car (exploratory analysis) separated by Task type*

**
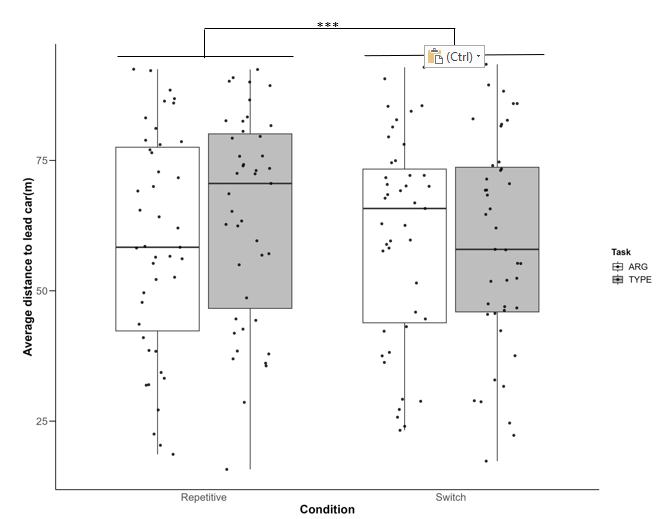
**

*Note:* Distribution of behavioral performance for each condition per task type with individual data points for Average distance to lead car

**Figure B2**

*Average distance to lead car (exploratory analysis) separated by Modality*

**
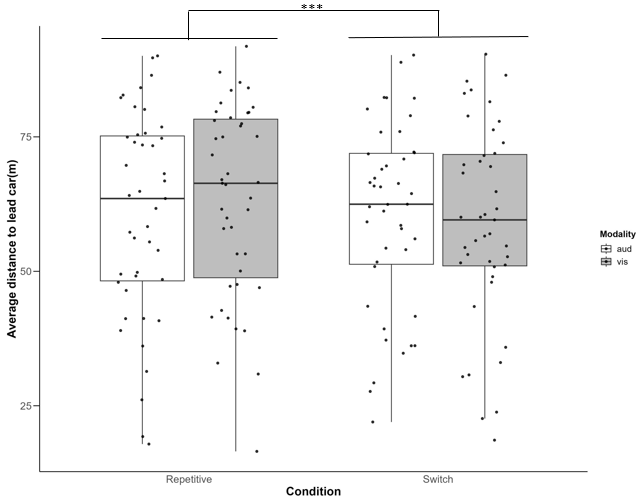
**

*Note:* Distribution of behavioral performance for each condition per task modality with individual data points for Average distance to lead car

**Figure B3**

*Standard deviation of lateral position*


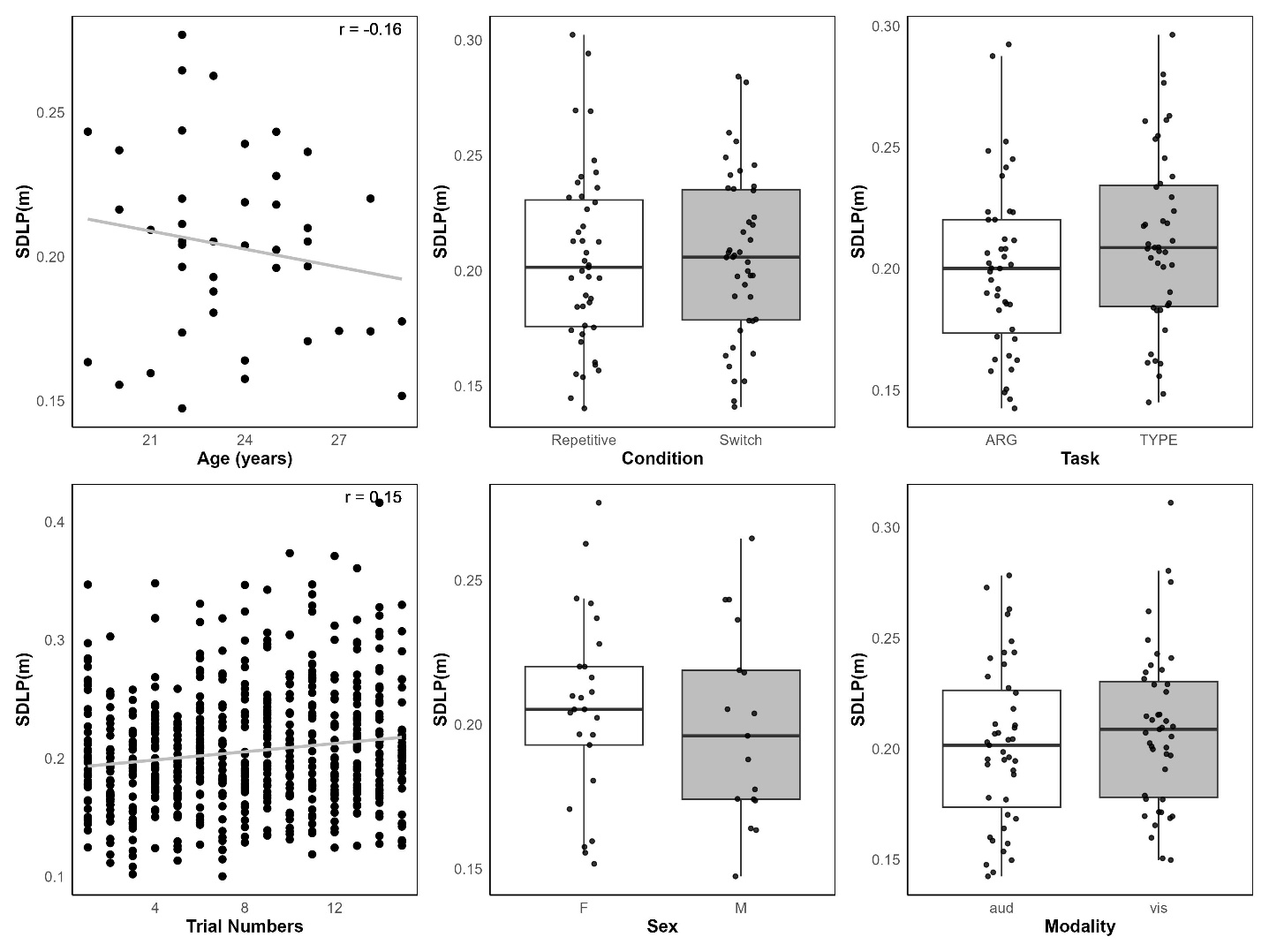


*Note.* Scatter plots and box plots are presented for main effect and covariates. The scatter plot shows the relationship between Age and Standard Deviation of Lateral Position (SDLP) and Trial number and Standard Deviation of Lateral Position. The box plots show Standard Deviation of Lateral Position distribution for Condition, Sex, Modality and Task.

**Figure B4**

*Average Velocity*


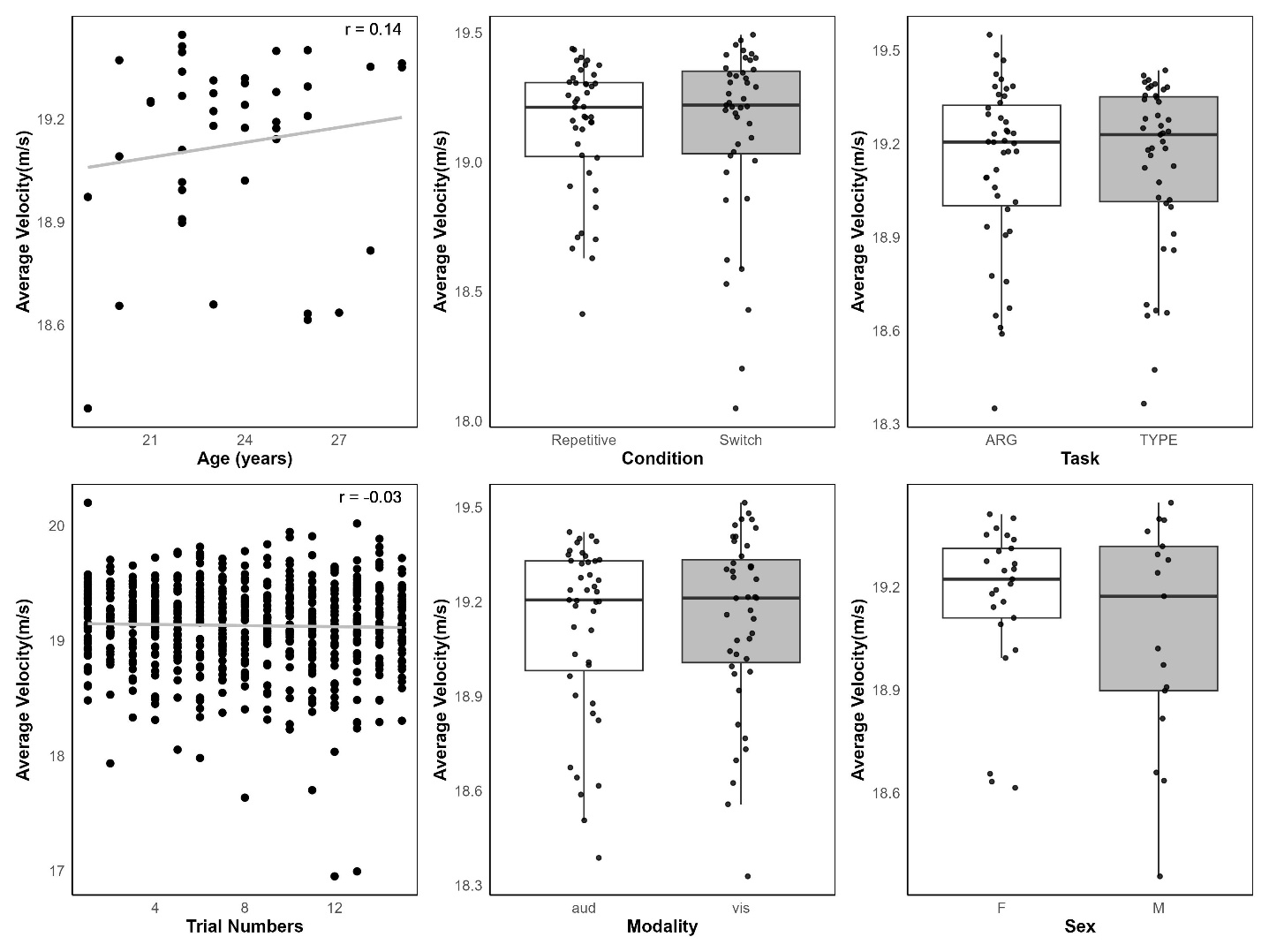


*Note.* Scatter plots and box plots are presented for main effect and covariates. The scatter plot shows the relationship between Age and Average Velocity and Trial number and Average velocity. The box plots show Average Velocity distribution for Condition, Sex, Modality and Task.

**Figure B5**

*Reaction Time of additional tasks*


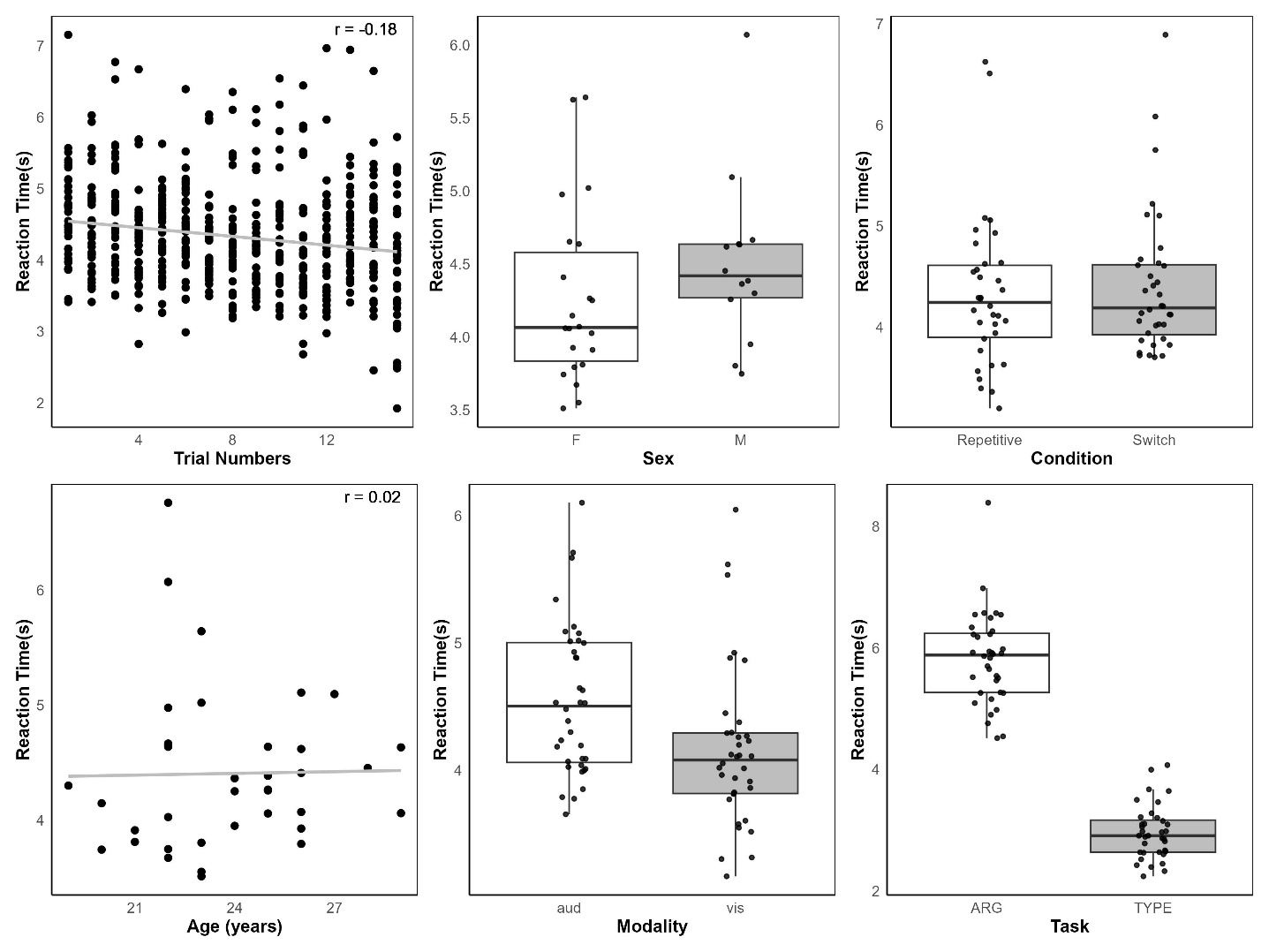


*Note.* Scatter plots and box plots are presented for main effect and covariates. The scatter plot shows the relationship between Age and Reaction Time of additonal tasks and Trial number and Reaction Time of additional tasks. The box plots show Reaction Time for Condition, Sex, Modality and Task.

**Figure B6**

*Standard deviation of average velocity*

**
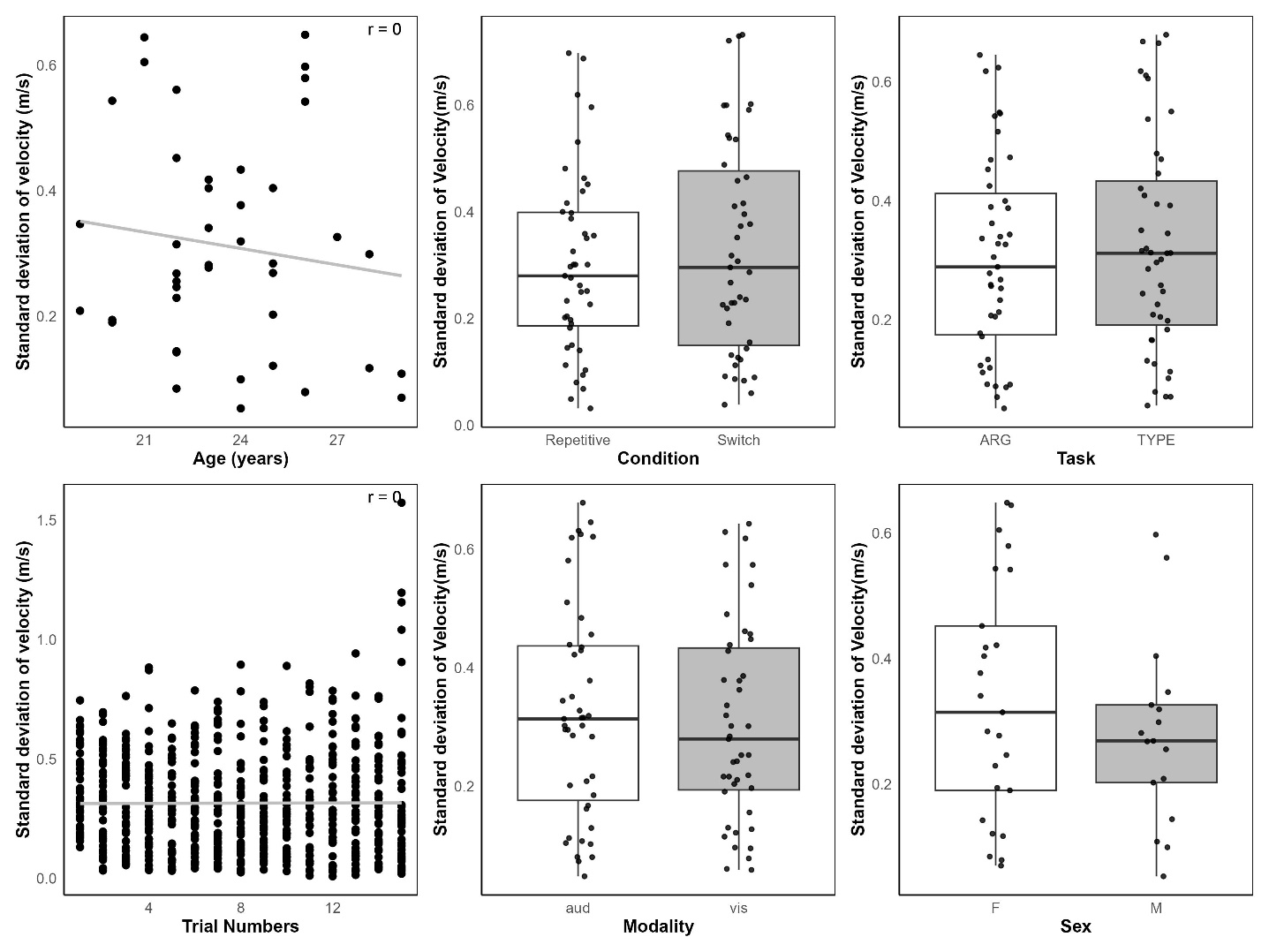
**

*Note.* Scatter plots and box plots are presented for main effect and covariates. The scatter plot shows the relationship between Age and Standard deviation of Average Velocity and Trial number and Standard Deviation of Average Velocity. The box plots show Standard deviation of Average Velocity distribution for Condition, Sex, Modality and Task.

**Figure B7**

*Average distance to lead car*

**
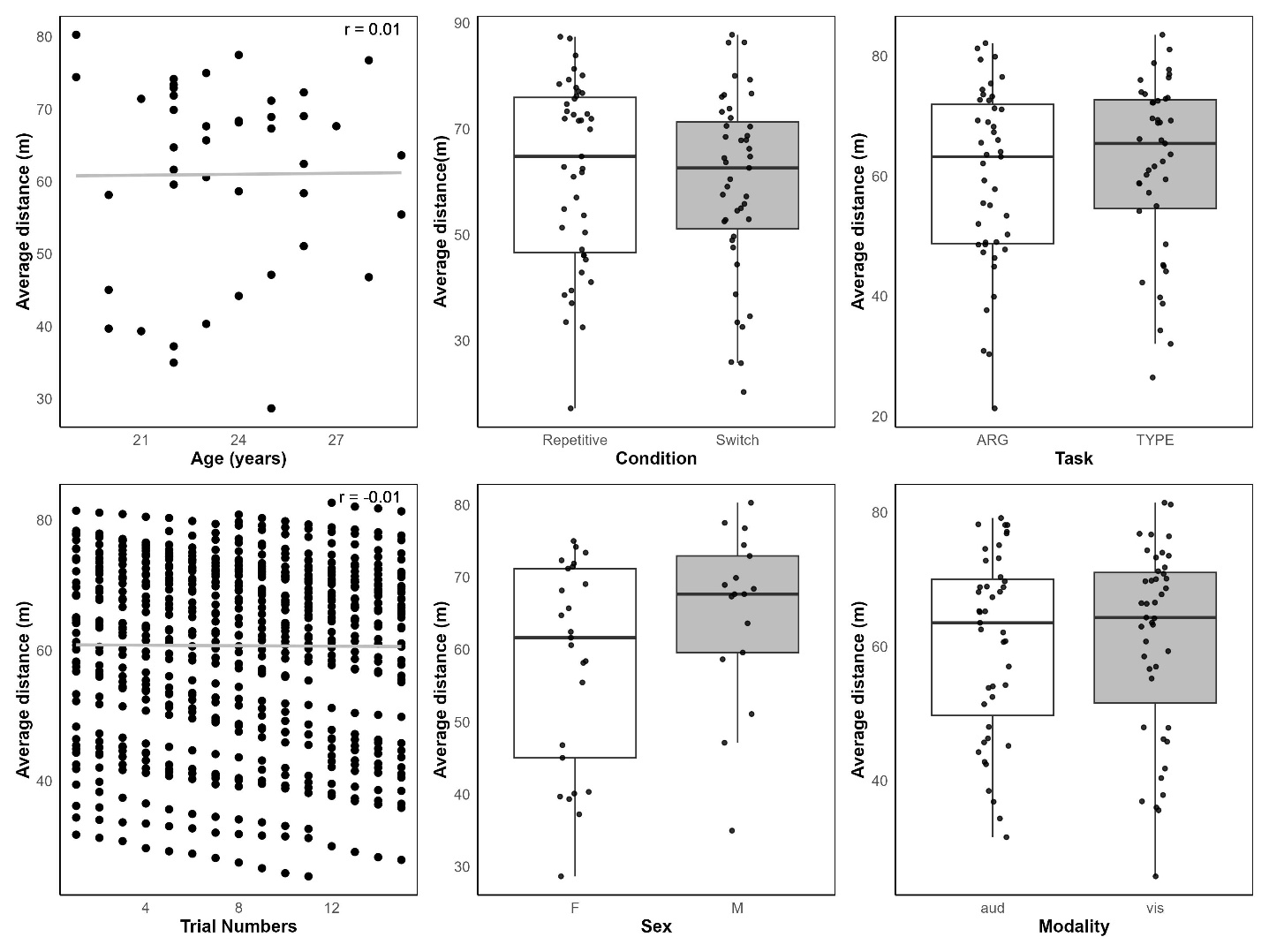
**

*Note.* Scatter plots and box plots are presented for main effect and covariates. The scatter plot shows the relationship between Age and Average distance to lead car and Trial number and Average distance to lead car. The box plots show Average distance to lead car distribution for Condition, Sex, Modality and Task.

**Figure B8**

*Standard deviation of average distance to lead car*

**
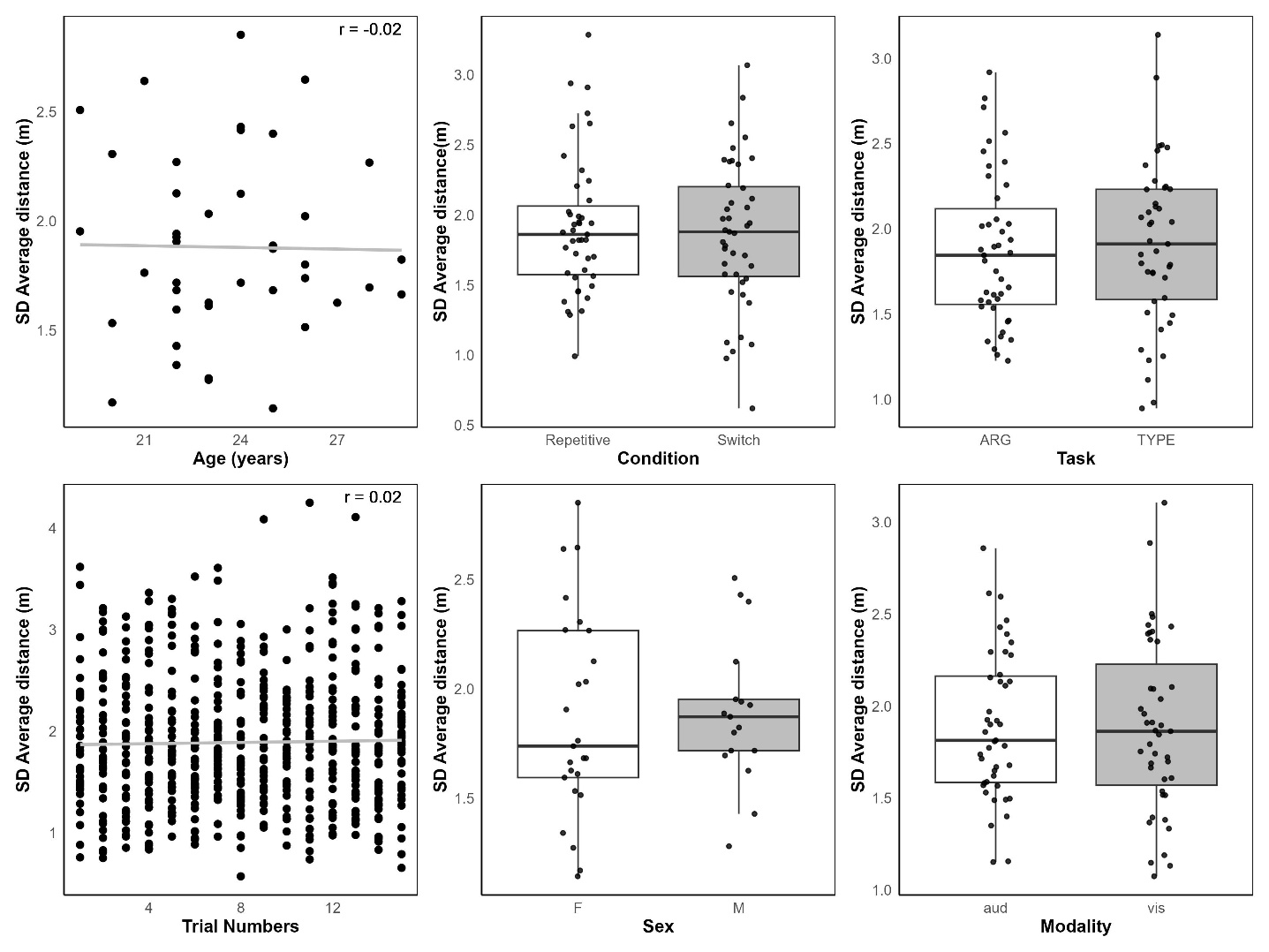
**

*Note.* Scatter plots and box plots are presented for main effect and covariates. The scatter plot shows the relationship between Age and Standard Deviation of Average distance to lead car and Trial number and Standard Deviation of Average distance to lead car. The box plots show Standard Deviation of Average distance to lead car distribution for Condition, Sex, Modality and Task.

**Figure B9**

*Average lateral position*

*
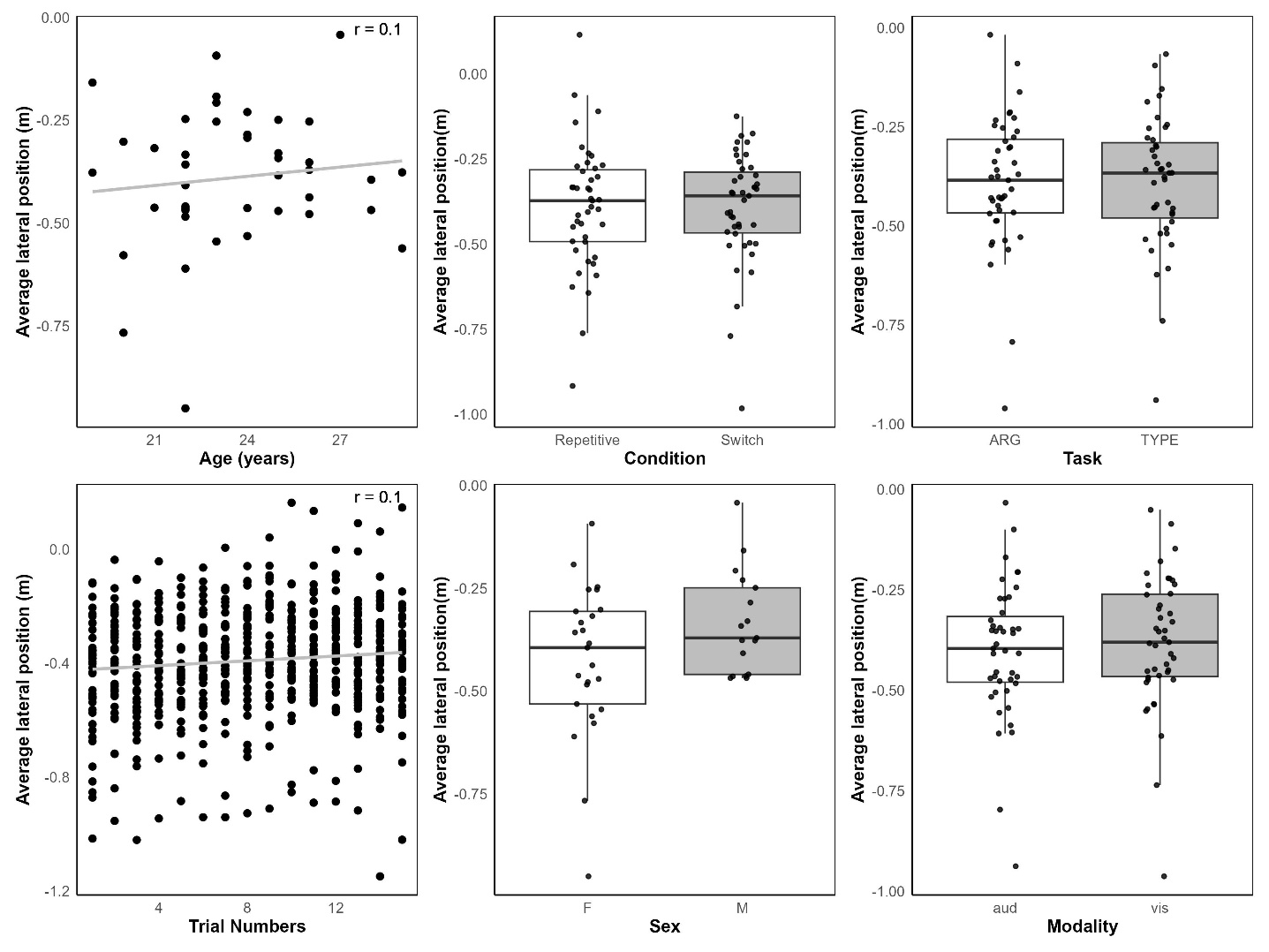
*

*Note.* Scatter plots and box plots are presented for main effect and covariates. The scatter plot shows the relationship between Age and Average Lateral Position and Trial number and Average Lateral Position. The box plots show Average Lateral Position distribution for Condition, Sex, Modality and Task.

# **Appendix C**

# **Post-hoc Results for Average Distance to Lead Car**

# **Findings from the exploratory analysis for Average distance to lead car**

As Condition * Task and Condition * Modality were significant, we performed a planned Turkey's HSD post hoc test. For Condition*Task, the post hoc test showed no significant difference between conditions during the Type task (β = 5.52, p = .10, ηG² = 0.01) or during the ARG task (β = -1.10, p = .74, ηG² = 0.01). Further, for Condition * Modality, the post hoc test showed no significant difference between conditions during the visually presented tasks (β = 4.37, p = .19, ηG² = 0.01) or during the auditory presented tasks (β = 0.04, p = .99, ηG² = 0.01).
